# Morphea: The 2023 update

**DOI:** 10.3389/fmed.2023.1108623

**Published:** 2023-02-13

**Authors:** Cristian Papara, David A. De Luca, Katja Bieber, Artem Vorobyev, Ralf J. Ludwig

**Affiliations:** ^1^Department of Dermatology, University of Lübeck, Lübeck, Germany; ^2^Lübeck Institute of Experimental Dermatology (LIED), University of Lübeck, Lübeck, Germany

**Keywords:** morphea, localized scleroderma, diagnosis, pathogenesis, treatment

## Abstract

Morphea, also known as localized scleroderma, is a chronic inflammatory connective tissue disorder with variable clinical presentations, that affects both adults and children. It is characterized by inflammation and fibrosis of the skin and underlying soft tissue, in certain cases even of the surrounding structures such as fascia, muscle, bone and central nervous system. While the etiology is still unknown, many factors may contribute to disease development, including genetic predisposition, vascular dysregulation, T_H_1/T_H_2 imbalance with chemokines and cytokines associated with interferon-γ and profibrotic pathways as well as certain environmental factors. Since the disease may progress to permanent cosmetic and functional sequelae, it is crucial to properly assess the disease activity and to initiate promptly the adequate treatment, thus preventing subsequent damage. The mainstay of treatment is based on corticosteroids and methotrexate. These, however, are limited by their toxicity, especially if applied long-term. Furthermore, corticosteroids and methotrexate often do not sufficiently control the disease and/or the frequent relapses of morphea. This review presents the current understanding of morphea by discussing its epidemiology, diagnosis, management and prognosis. In addition, it will describe recent pathogenetic findings, thus proposing potential novel targets for therapeutic development in morphea.

## 1. Introduction

Morphea, also known as localized scleroderma, is a rare inflammatory connective tissue disorder occurring primarily in children aged 2–14 years (1, 2), and in women (3). It is characterized by inflammatory patches and/or bands of thickened skin on the head and neck region, trunk and extremities (4). Depending on the extent and depth of fibrosis, it is classified into five main types (limited, generalized, linear, deep and mixed) as well as various subtypes (plaque-type, pansclerotic, *en coup de sabre*, etc.) (5). Even though it is considered a skin-limited disease, certain subtypes are associated with extracutaneous manifestations, such as musculo-articular (myositis, fasciitis and arthritis), central nervous system (headache, migraine, seizures, and epilepsy) and ocular (uveitis) (4). In addition, they may lead to severe disfigurement (residual hyperpigmentation and skin atrophy), functional disability (joint contractures) and neuro-ophthalmologic complications (6, 7). Despite the presence of extracutaneous manifestations, morphea must be distinguished from systemic sclerosis (SSc) (4). It is, nevertheless, noteworthy to stress that morphea does not transit to SSc.

While the exact cause of the disease is still not known, certain stimuli (infection, drugs and/or trauma) may trigger vascular and immune dysregulations in genetically predisposed individuals. Particularly T-cell activation and the release of cytokines associated with interferon-γ (IFN-γ) are involved, thus leading to the activation of inflammatory and profibrotic pathways that result in excessive collagen production (6, 8–11).

To date, there is no cure for morphea and therapy remains a major clinical challenge. Depending on the disease type, extent, severity, and extracutaneous involvement, treatment options are classified into general non-pharmacological measures, topical and systemic treatment (12). The current therapeutic options are, however, limited, not disease-specific and their long-term use is often associated with several adverse events. Furthermore, the disease is characterized by a chronic, relapsing–remitting course, and the presence of atrophy and extracutaneous complications may lead to significant cosmetic, physical, functional, and mental disabilities (13–17).

Herein, we review the various clinical presentations of morphea, the most recent advances regarding its pathogenesis, as well as the many challenges that the clinicians encounter in disease diagnosis, severity assessment and appropriate treatment selection.

## 2. Epidemiology

Morphea is a rare inflammatory connective tissue disease, with a total annual incidence ranging from 4 to 27 new cases per million people (18, 19). Nearly two-thirds of all cases occur in adults, whereas juvenile localized scleroderma was estimated to have an annual incidence rate of 3.4–9 cases per million children per year (2, 18, 20, 21). Of note, morphea is about 6–10 times more common in children than SSc, while in adults the annual incidence rates were similar or even higher in SSc (21–23).

Two incidence peaks of morphea are observed: one between 2 and 14 years, and a second one in the fifth decade of life (1, 2). The reported mean ages of disease onset for juvenile and adult morphea were 10 and 45 years, respectively (24). Moreover, the disease exhibits a female preponderance with an overall female-to-male ratio of 4:1 (1, 2, 21). Even though it may occur in all races, Caucasians seem to be the most affected by the disease, followed by Hispanic and Latin American patients (1, 2, 25).

The most common variant of morphea in adults is the plaque-type, followed by the generalized variant, whereas in children the linear form is the most prevalent (1, 2, 18, 26). A family history for connective tissue or autoimmune diseases in first- and second-degree relatives is seen in 22% of children and 11% of adults diagnosed with morphea (2). Remarkably, the generalized and mixed types have the highest association with familial autoimmune diseases.

An uncommon and underestimated disease variant is the congenital localized scleroderma, which is characterized by a mean diagnosis delay of 3.9 years (27). In a demographic study among juvenile localized scleroderma patients, skin lesions were observed in 0.8% of cases at birth and the female-to-male ratio for this disease type was 2:1 (27). The most common clinical presentation was the *en coup de sabre* subtype (27–29).

## 3. Pathogenesis

**Figure 1:**
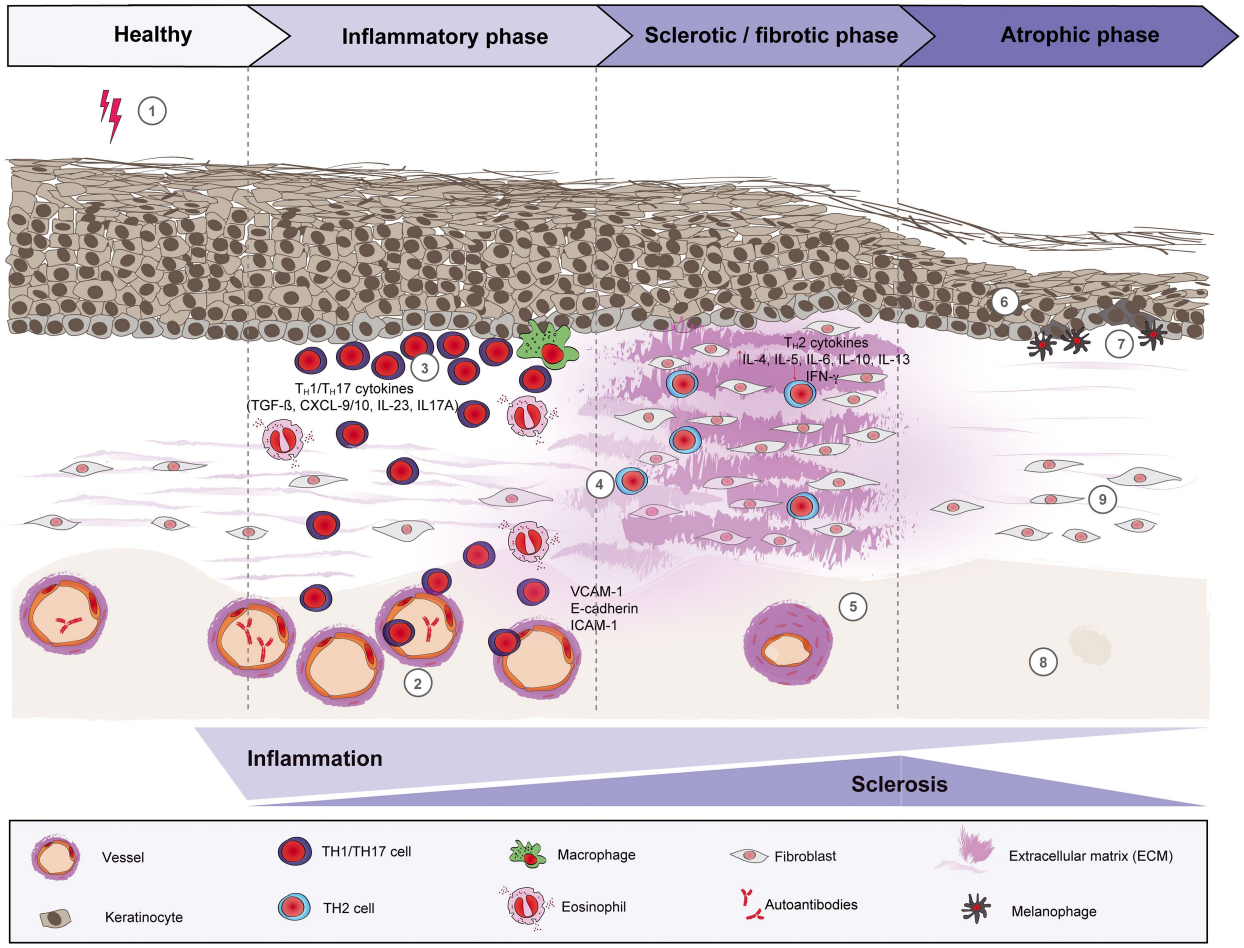
Schematic overview of morphea pathogenesis. Based on current evidence, the pathogenesis of morphea can be divided into three distinct phases: early inflammatory, sclerotic/fibrotic and late atrophic. Environmental factors like radiation, skin trauma and infections may trigger in genetically predisposed patients (1) a T cell-driven skin inflammation, but also plasma cells and eosinophils around the vessels, adnexal structures and in the dermis. The resulted intense endothelial damage will lead to the upregulation of adhesion molecules, such as E-cadherin and VCAM-1 during the inflammatory stage (2), which in turn will recruit pro-inflammatory TH1 and TH17 cells and associated cytokines (CXCL-9/10, TGF-ß, IL-23 and IL-17A, respectively) that will activate fibroblasts. (3) Next, a switch towards a predominant TH2-driven response will facilitate the recruitment of T lymphocytes that are capable of producing profibrotic cytokines like IL-4, IL-6 and TGF-ß. (4) Therefore, sclerosis increases with hyalinized, compact collagen bundles in the dermis, with few sweat glands and blood vessels, the latter with thickened walls and narrow lumens. (5) In the last phase, atrophy slowly increases as sclerosis subsides. The epidermis will decrease in thickness (6), while basal keratinocytes display pigment, with the presence of underlying melanophages. (7) There is loss of skin appendages, blood vessels (8) and inflammatory cells (9).

The pathogenesis of morphea is still not very well understood. A variety of factors, including genetics, environmental factors, such as infections, skin trauma, autoimmune dysregulation with abnormal cytokine production, and/or vascular dysfunction may play a role in the development of morphea. In general, three phases can be distinguished: (i) an early inflammatory phase, (ii) a fibrotic/sclerotic phase, and (iii) an atrophic phase (Figure 1).

### 3.1. Genetics and environmental factors

Putative genetic associations of morphea include HLA class I and II genes. The strongest associations were found with DRB1*04:04 and HLA-B*37. The morphea-associated alleles are different from those found in SSc, suggesting that morphea is immunogenetically distinct (30–32). Individuals with morphea have a higher frequency of concomitant and familial autoimmunity. Alleles associated with morphea are in parallel strongly associated with rheumatoid arthritis (RA), autoimmune thyroid disease (AITD), multiple sclerosis (MS) and type 1 diabetes mellitus. Interestingly, population-based studies observing the autoimmune profile of RA, MS, and AITD have identified an increased risk of morphea in these patients, suggesting a common genetic susceptibility (31). In addition, high prevalence of concomitant and familial autoimmune diseases was found (2). Furthermore, up to 50% of patients have elevated levels of three main autoantibodies: antinuclear (ANA), anti-histone (AHA), and anti-single-stranded DNA (ssDNA) antibodies, whereas other autoantibodies are observed at frequencies below 10%, indicating that morphea involves autoimmune abnormalities against an unknown self-antigen (2, 33, 34).

As generalized scleroderma has been linked to other genes beside the HLA loci that are also involved in pathogenesis of scleroderma like transforming growth factor-ß (TGF-ß) and monocyte chemoattractant protein-1 (MCP-1), it is likely that these genes could be also linked to morphea but further investigations are still needed (4). Beside the genetic component, various exogenous triggers are suggested, including some infections as for SSc, *Epstein–Barr virus*, *varicella zoster virus* and *Borrelia burgdorferi*, as well as local trauma, surgical operations, radiation and BCG vaccinations or adjuvants (6, 35–37). Case reports concerning an effect of COVID-19 infections as well as vaccinations were published but the data have to be confirmed (38–41).

### 3.2. The early inflammatory phase

In the early stage of morphea, a large number of mononuclear lymphocytes (primarily activated T lymphocytes but also macrophages), some plasma cells and eosinophils infiltrate the skin and surrounding blood vessels (42–44). Preferentially CD4+ lymphocytes and their associated cytokine and chemokine profiles are observed in both blood and skin, whereas the number of functional T regulatory cells is decreased (11, 45). Here, it is shown that the release of a large amounts of cytokines by lymphocytes occurs before as well as after cellular activation and invasion. These cytokines probably determine the direction of the immune response and control the disease severity. Less data is available on cytokine profiles in skin lesions, but recently published juvenile morphea gene signatures compared to healthy controls showed an inflammatory response gene signature composed of IFN-γ-, IFN-α-, and tumor necrosis factor-α-associated genes like chemokine (C-X-C motif) ligand (CXCL)-9, CXCL-10, CXCL-11 and IFN-γ itself in correlation with the clinical disease activity (45, 46).

Interestingly, earlier publications indicate mostly cytokines associated with T helper 2 (T_H_2) immune responses, such as interleukin (IL)-4 and IL-6 at increased levels in serum of patients with morphea. Specifically, IL-4 and IL-6 were increased by 17 and 47%, respectively, in the serum of patients with morphea in contrast to healthy controls (9). In contrast, immunohistochemical analysis showed the expression of all, T_H_1 (CD4 and T-bet), T_H_2 (CD4 and GATA-3), T_H_22 (CD4 and BNC-2) and T_H_17 [CD4 and signal transducer and activator of transcription (STAT)-3] cell markers in morphea skin lesions (47). The literature available, however, does not examine the presence or elevation of T_H_ effector subsets in reference to early or late disease.

Based on functional *in vitro* data and cytokine analysis (mostly in the serum of patients) it is suggested that a T_H_1/T_H_2 imbalance in morphea is propagating the disease (48–50). There is an overall notion that pro-inflammatory T_H_1/T_H_17-associated cytokines are elevated during the early stages. During the progression of the disease a shift towards T_H_2 cytokines (IL-4, IL-5, IL-6, IL-10, and IL-13) is postulated, leading to skin fibrosis and damage. IL-4 produced by CD4+ T_H_2 lymphocytes can upregulate the production of TGF-ß by T lymphocytes and other cells (9, 42, 48, 51). Of note, both IL-4 and TGF-β increase collagen synthesis, and IL-4 furthermore promotes fibroblast proliferation. In addition, TGF-ß is capable of stimulating fibroblast production of type I collagen, type III collagen and other extracellular matrix proteins (42, 52). This shift to a T_H_2 signature could potentially induce the development of tissue damage and fibrosis later in the course of the disease.

The inflammatory phase is accompanied by changes in the vascular endothelium (and lymphatic vessels) (6, 53). Up-regulation of several adhesion molecules, such as soluble E-selectin and soluble vascular adhesion molecule-1 (sVCAM-1) was observed in sera of morphea patients (6, 54) and upregulation of VCAM-1 was observed also in skin lesions (55). These adhesion molecules are important for the adherence of monocytes to the endothelium and the recruitment to the area of inflammation, as they facilitate the processes of rolling, adhesion and transmigration (56).

### 3.3. Fibroblast activation and the sclerotic phase

It is postulated that injury of the vascular endothelium and upregulation of adhesion molecules, such as E-selectin and VCAM-1 during the inflammatory stage facilitate the recruitment of T lymphocytes that are capable of producing profibrotic cytokines like IL-4, IL-6 and TGF-ß (6, 42). Fibrosis plays a critical role in causing tissue damage in scleroderma and is accompanied by hardening of the skin from excessive cellular proliferation as well as deposition of collagen and other extracellular matrix components. Upon tissue injury, fibroblasts differentiate into activated fibroblasts or myofibroblasts, the latter expressing smooth muscle actin unlike fibroblasts, and thus participate in wound healing processes. After the process, myofibroblasts are normally lost from the site of injury, whereas in fibrotic pathologies such as scleroderma they persist and play a major role in abnormal fibrotic pathologies (57). *In vitro* experiments have shown that tissue fibrosis is caused by overshooting TGF-β and IL-4 activity. TGF-β induces mitogenic activity in fibroblasts by matrix metalloproteinase (MMP)-3 and platelet-derived growth factor (PDGF) as well as the synthesis of several extracellular matrix proteins, such as collagens, fibronectin and others. Additionally, TGF-β blocks the collagenase synthesis (58). The profibrotic activity of IL-4 results in increased production of extracellular matrix proteins like collagen. Additionally, IL-4 has been shown to block IFN-γ, a cytokine that is secreted by activated T cells and known to be an inhibitor of procollagen synthesis in fibroblasts (59). Moreover, IFN-γ directly stimulates prostaglandin production (another fibroblast growth inhibitor) in monocytes (42).

Beside the fibrosis, altered distribution of CD34+ dermal dendritic cells (DCs) and further vascular abnormalities have been reported in relation to the sclerotic phase of morphea. CD34 stromal expression was significantly lower in morphea patients than in healthy controls (55). Studies on the involvement of individual DC subpopulations in the development of inflammatory infiltrates in morphea are still outstanding. Individual investigations demonstrated high numbers of plasmacytoid DC in skin lesions within deeper dermal layers, around blood vessels and around collagen fibers in subcutaneous tissue. The most numerous populations of DCs are myeloid DCs, which colonize almost all non-lymphoid peripheral tissues. They are thought to play a significant role in both the development of immune tolerance mechanisms and the activation of autoreactive T cells (60).

### 3.4. Atrophy occurs in late-stage lesions in morphea

Atrophy is a poorly understood pathogenic event that may persist long after the sclerotic phase of morphea. It may happen that sclerosis improves slowly (over 2–5 years), often after discontinuation of treatment but atrophy increased slightly as sclerosis subsided. Typical symptoms are focal atrophic epidermal changes, dense dermal collagen, few pigmented dermal macrophages, post-inflammatory hyperpigmentation and mild chronic inflammation. It often also affects the subcutis, bones and fat tissue (15). However, further information in morphea is limited and most information is based only on Ssc. Here, it has been shown that once the inflammatory reaction subsides, the disease burns out. Atrophy and long-term remodeling involving modified matrix-metalloproteinase profiles stimulated by T lymphocytes resolve tissue fibrosis (61).

## 4. Clinical types

Morphea can exhibit different clinical presentations, yet there is no consensus on the proper classification method (6). According to Kreuter et al. (5, 62), morphea can be divided into five main types, i.e., limited, generalized, linear, deep and mixed, the latter being a combination of at least two of the previous types. Each of these types may also have various subtypes (Table 1).

**Table 1:** Classification of morphea according to the German guideline by Kreuter et al. (5).

Morphea type	Morphea subtypes
Limited	
	Plaque-type morphea
	Guttate morphea
	Atrophoderma of Pasini and Pierini
Generalized	
	Generalized morphea
	Disabling pansclerotic morphea
	Eosinophilic fasciitis (Shulman syndrome)
Linear	
	Morphea of the extremities
	Morphea *en coup de sabre*
	Progressive facial hemiatrophy (Parry-Romberg syndrome)
Deep	
Mixed	
	Combination of the above-mentioned types

### 4.1. Limited type

This clinical form of morphea may present as classical plaque, guttate or superficial morphea, the latter also known as atrophoderma idiopathica of Pasini and Pierini.

**Figure 2:**
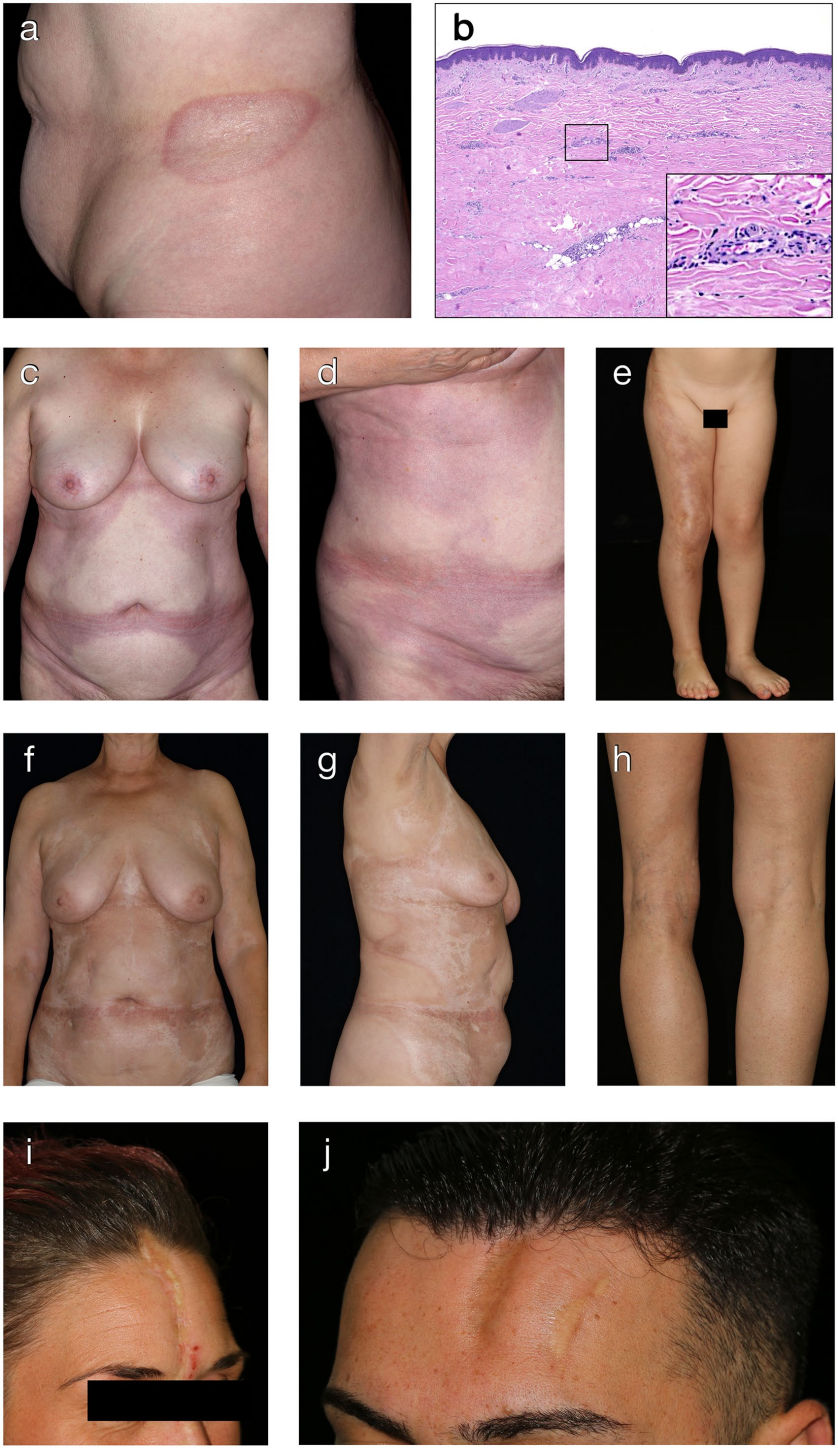
Clinical and histological hallmarks of morphea. **(A)** Well-defined oval patch with a central ivory white area surrounded by an erythematous violaceous rim (“lilac ring”) in a patient with plaque-type morphea. **(B)** The histology from a cutaneous biopsy from a sclerotic morphea lesion typically showing a thin epidermis, basal keratinocytes displaying pigment, scarce lymphocytic inflammatory infiltrates in the papillary dermis and around the vessels, with significant sclerosis in the reticular dermis and atrophy of the adnexal structures (H&E staining, 40×). Magnification displaying minimal periadnexal lymphocytic inflammatory infiltrates in the papillary dermis and thick, hyalinized, eosinophilic collagen bundles in the reticular dermis with entrapped atrophic adnexal structures (H&E staining, 200×). **(C,D)** Extensive, well-demarcated, coalescing erythematous violaceous patches on the trunk indicative of an early, inflammatory stage of generalized morphea. **(E)** Band-like, atrophic, hyperpigmented plaque in a blaschko-linear distribution in a child with linear morphea of the limb. **(F,G)** Ill-defined, coalescing, pink erythematous patches with important central sclerosis in a patient with generalized morphea, sclerotic disease stage. **(H)** Atrophy of the underlying tissue with asymmetry of the limbs in a patient with deep morphea. **(I,J)** Median and paramedian linear depressed, sclerotic plaques of morphea *en coup de sabre* with the presence of cicatricial alopecia.

Plaque-morphea is the most common form of localized scleroderma in adults (5, 18). It is characterized by round- or oval-shaped, brownish or yellow-whitish plaques localized in one or two anatomical sites, such as back, upper and lower extremities, buttocks, face, neck or scalp (63, 64). Commonly affected areas include the submammary region, groin and lower abdomen. During the initial phase, round- or oval-shaped, rather erythematous and/or edematous plaques expand centrifugally leaving a slight induration in the center of the lesion (Figure 2A). Active plaques are often surrounded by a violaceous halo (“lilac ring”), denoting the inflammatory disease stage (65). With further disease progression, the central induration increases, the lesion turns sclerotic with a whitish or ivory colored, shiny surface. After a disease activity of months to years, lesions become less sclerotic and more atrophic, showing a fine, wrinkled skin with dyspigmentation and loss of skin appendages.

Guttate morphea is a superficial progressive variant with a self-limited course. In early stages, the disease is characterized by the presence of initially erythematous and ultimately yellowish macules. In addition, multiple small shiny infiltrated plaques of up to 10 mm can be typically found on the trunk. Generalized, disseminated variants have also been reported (5, 66).

Atrophoderma idiopathica of Pasini and Pierini or superficial morphea, is a rare condition with initially asymptomatic lesions that usually begin on the back and then progress to the trunk and arms, eventually leading to pain and pruritus. Symmetrical, hyperpigmented, round, slightly depressed and sharply demarcated plaques with varying diameters, from millimeters to several centimeters, are characteristic. Of note, the “lilac ring” is absent and the skin is usually not indurated (5, 67).

In addition to the above-mentioned subtypes, other forms, such as bullous and keloidal/nodular morphea have also been described (68–70). Bullous morphea is an extremely rare variant of localized scleroderma, usually affecting the lower extremities. Typically, it presents with tense subepidermal bullae associated with characteristic morphea lesions. Blistering is considered to be the result of lymphatic obstruction caused by skin sclerosis (70–72).

### 4.2. Generalized type

Generalized morphea (Figures 2C,D,F,G) is characterized by multiple, coalescing, indurated plaques, that are predominantly found on the trunk, legs and lumbosacral region, in a usually symmetrical distribution (73). It is defined by the presence of at least four lesions, more than 3 cm in diameter and affecting two or more anatomic sites. Generalized morphea should not present signs of SSc, such as Raynaud phenomenon, sclerodactyly, capillaroscopy abnormalities as well as specific autoantibodies (74).

A rare variant of generalized morphea is the disabling pansclerotic morphea, which is characterized by widespread, circumferential skin distribution, sparing the fingers and toes as well as significant subcutaneous tissue, fascia, muscle and bone involvement (75, 76). As a result, contractures, large ulcerations and impaired wound healing are frequently described in association with this subtype. The disease usually starts in childhood and it is associated with important mortality, death causes including sepsis, gangrene and cardiopulmonary disease (76, 77).

Eosinophilic fasciitis or Schulman syndrome is a disease with acute onset that presents with painful, symmetrical swelling, typically on the extremities, however, without the involvement of hands and feet. In later stages, a deep sclerosis replaces the edema conferring a typical “peau d’orange” appearance (78). Characteristic is the negative vein sign, also known as “the groove sign,” which is a depression along the course of the superficial veins demarcated by the surrounding tissue (79). Eosinophilic fasciitis may be considered a form of localized scleroderma, since it can exhibit concomitant morphea lesions in approximately 40% of patients (80).

### 4.3. Linear type

Linear scleroderma is characterized by linear or band-like localized lesions that can affect the dermis, subcutaneous tissue, muscles as well as underlying bones. It represents the most frequent form of localized scleroderma in children and adolescents, namely 40–65% of juvenile morphea (1, 2, 18). Linear morphea often occurs as a single unilateral lesion on the extremities (Figure 2E) or face and scalp, and it often follows the lines of Blaschko (81). These lesions may heal with hyperpigmentation or may cause growth retardation, muscle atrophy and myositis. If linear morphea lesions are present over the joints, it can result in flexion contractures and severe movement impairment, respectively. In some cases, growth defects of underlying muscles and bones leading to limb-length discrepancies can also occur (82).

Linear morphea of the head/face and neck area causes an atrophic depression of the skin, often involving also the underlying soft tissue, bone and brain, characteristically resembling the cut from a sword, therefore also known as linear morphea *en coup de sabre* (Figures 2I,J). The lesion is usually unilateral and mostly affects the frontoparietal region or the paramedian forehead. If the scalp is involved, it leads to irreversible, scarring alopecia (Figure 2I). The association of morphea *en coup de sabre* with neurologic manifestations, such as seizures, headaches, facial paralysis and neuropathy, has been reported (1, 83–85).

Parry-Romberg syndrome, also known as progressive facial hemiatrophy, is another variant of linear morphea of the head and face, which is characterized by unilateral atrophy of underlying soft tissue and bone, but not the superficial skin (86). It usually appears during the first two decades of life and more frequently in girls. The disease may often coexist with morphea *en coup de sabre*, some data even suggesting that it might be the severe variant of the latter (86, 87). Palate, gingiva and tongue may also be affected (88). In some cases, involvement of central nervous system (CNS) has been described (89).

### 4.4. Deep type

Deep morphea, also known as “solitary morphea profunda,” is a rare variant of localized scleroderma that manifests as a sclerotic process affecting the deep reticular dermis, subcutaneous fat tissue and, in some cases, the underlying fascia (Figure 2H). Typically, a single lesion on the upper back or extremities is observed (90, 91). However, generalized forms may also occur exceptionally (92). In certain cases, injection or trauma prior to disease manifestation have been identified as potential triggers (92–94).

### 4.5. Mixed type

Mixed morphea represents the combination of at least two of the above-described types. It is encountered in approximately 15% of juvenile morphea patients and the most frequent association is the limited and linear type (1).

## 5. Diagnosis

Morphea exhibits a broad spectrum of clinical presentations. Even though these clinical variants are well characterized, the disease is often under-diagnosed or mistaken for other dermatological diseases, particularly in the early stages. This may result in a delay of diagnosis of several months to years (20). In addition, in congenital morphea the disease duration until appropriate systemic treatment initiation was found to be even longer, i.e., up to 4 years, hence a greater risk for physical and cosmetic sequalae (27, 28).

### 5.1. Histopathology

The diagnosis of morphea is mainly based on clinical features. A skin biopsy for histopathological evaluation is usually reserved for atypical, doubtful cases. The biopsy has to be sufficiently deep, since some types of morphea affect the subcutis or underlying fascia and muscle (4). However, there are no specific histopathology features for morphea and routine histopathology can neither differentiate among the various types nor to distinguish it from SSc (95). Still, it can provide details regarding the disease state. Early inflammatory skin lesions show: (i) thick collagen bundles in the reticular dermis that run parallel to the skin surface, (ii) dense inflammatory infiltrates comprising lymphocytes, eosinophils, plasma cells and histiocytes between the collagen bundles, in the perivascular and periadnexal areas, (iii) normal or atrophic overlying epidermis. Later fibrotic skin lesions become less inflammatory, avascular with thickened blood vessel walls and narrow lumens, and collagen bundles get thick, compact, and highly eosinophilic with few or absent sweat glands (Figure 2B). In addition, collagen may replace the underlying subcutaneous tissue.

### 5.2. Laboratory tests

Even if there are currently no diagnostic tests available for morphea, baseline investigations are recommended, in particular if systemic treatment is being considered. These should include full blood count, kidney and liver function tests, creatine kinase (in case of suspected concomitant myositis), rheumatoid factor (in case of suspected concomitant arthritis) and C-reactive protein (CRP) (95). Hypergammaglobulinemia, elevated CRP and eosinophilia are found in the active stages of linear morphea, while the latter is also seen in the generalized type (25, 96–98). Elevated creatine kinase was associated with muscle atrophy and extremity shortening, while raised aldolase was linked to joint contractures (99).

Many autoantibodies have been detected in morphea, including ANA, ssDNA and AHA antibodies (33). Positive ANA titers were found in up to 70% of morphea patients, being associated with a higher risk for extracutaneous, deeper involvement and disease relapse (1, 2, 16, 26, 96, 100). ssDNA and AHA antibodies were linked to joint and muscle involvement as well as a higher number of skin lesions, and in certain patients even to disease activity (34, 96, 101). IgA, IgM and IgG levels were found to be increased in linear, deep and pansclerotic morphea (1). Routine antibody screening is, however, not currently recommended. Likewise, screening for specific SSc antibodies and routine PCR-based or serological testing for *Borrelia burgdorferi* should not be performed, apart from high clinically suspicious cases (95).

### 5.3. Imaging

#### 5.3.1. Magnetic resonance imaging

MRI of the brain with contrast is recommended in all patients with morphea affecting the face, head and neck area, regardless of the presence of characteristic neurological symptoms (headaches/migraine, seizures, hemiparesis) (29, 95). Up to 50% of the patients with morphea lesions on the head/face do not exhibit any neurological symptoms, yet still have intracranial abnormalities on MRI (102). These may include white matter and leptomeningeal enhancements, dystrophic calcifications, sulcal crowding, cerebral atrophy and even CNS vasculitis (25, 83, 102–104). MRI scans should be done at baseline and repeated if any neurological symptoms develop during follow-up (105). MRI of the affected limb with contrast is indicated in all patients suffering from deep and/or extensive morphea of the extremities in order to detect any joint, muscle or bone involvement, or before planning plastic-surgical interventions.

#### 5.3.2. Ultrasonography

Ultrasonography, particular in the 20-MHz range, can be used to accurately assess the activity and damage of skin lesions in morphea, with results comparable to the clinical validated score localized scleroderma cutaneous assessment tool (LoSCAT) (62, 106–108). Early lesions are characterized by hypoechogenicity, whereas later fibrotic lesions are hyperechogenic (109). Color Doppler may also be helpful to identify disease activity, since active lesions are characterized by an increased blood flow and subcutaneous hyperechogenicity (110). Recently, Salgueiro et al. (111) proposed a novel diagnostic ultrasound sign for morphea, called the “sun sign”. This consists of a hyperechogenic halo surrounding superficial subcutaneous veins of the extremities in transverse view. Nevertheless, ultrasonography is not yet validated for routine use in the evaluation of morphea.

#### 5.3.3. Reflectance confocal microscopy and optical coherence tomography

Reflectance confocal microscopy (RCM) and optical coherence tomography (OCT) are non-invasive real-time imaging techniques that may aid in the diagnosis of various inflammatory skin diseases, including morphea (112–114). While RCM provides structural analysis of only the horizontal sections of the skin, OCT evaluates the morphology of both horizontal and vertical skin sections. In addition, high-definition OCT (HD-OCT) allows a more in-depth skin analysis, making it suitable for sclerodermiform conditions. In morphea, HD-OCT showed a uniform darkness (hyporefractiveness) in the dermis corresponding to the dermoscopic and histopathologic presence of sclerosis (115). In contrast, the inflammatory stage of morphea is characterized by large poorly backscattering dermal areas with fewer and smaller blood vessels in the affected area when compared to the normal surrounding tissue. In contrast, RCM showed pronounced hyperreflective areas with severe eccrine gland atrophy and no sebaceous glands in a patient with *en coup de sabre* morphea (116). In view of this, RCT and/or OCT may be used as ancillary diagnostic tools for morphea, allowing real-time examination of the skin, identification of appropriate biopsy site, thus hastening the diagnosis, reducing the delay of treatment initiation and improving patient management.

#### 5.3.4. Thermography

Infrared thermography may also aid in the identification of active morphea variants, with a sensitivity and specificity of 80.7 and 86.3%, respectively (117, 118). In addition, it was shown to positively correlate with the erythema and dermal atrophy clinical scores. Still, this method has many limitations and its use in clinical practice for morphea may not be feasible yet. Further studies testing infrared thermography in morphea patients are needed.

#### 5.3.5. Dermoscopy

The most characteristic dermoscopic features of morphea include whitish fibrotic beams, structureless or network-like pigment structures and linear branching vessels (119). These features correlated with the pathological modifications, thus making dermoscopy a reliable tool for the clinical diagnosis and management of morphea (119–121). In addition, it may allow its differentiation from other inflammatory skin disorders, mainly lichen sclerosus (121).

### 5.4. Measurement of disease activity and severity

The correct assessment of disease severity is a crucial step in the evaluation of all patients with morphea, in particular in pediatric variants. An incorrect severity assessment may lead to a delayed initiation of appropriate systemic treatment, and thus to irreversible physical and functional disabilities (7, 12). Moreover, pediatric morphea is associated with a more severe disease course and higher risk of relapse, the latter occurring even after years of remission (13, 14, 16, 17).

Localized scleroderma cutaneous assessment tool (LoSCAT; Table 2) is a scoring tool that assesses both disease activity and damage in morphea by combining the Physician’s Global Assessment (PGA), localized scleroderma skin severity index (LoSSI) and localized scleroderma skin damage index (LoSDI) scores (122–124). LoSSI is a validated skin score that measures the activity and severity of skin lesions in morphea by including four parameters (body surface area, erythema, skin thickness and development of new lesions or previous lesional extension) assessed in 18 anatomical regions and graded from 0 to 3 (122, 124). LoSDI measures skin damage by incorporating three parameters: atrophy, loss of subcutaneous tissue and hypo- or hyper-pigmentation (123).

**Table 2:** Differential diagnosis of morphea types.

Morphea type	Differential diagnoses
Limited morphea	
Early stage	Lichen sclerosus
	Granuloma annulare
	Cutaneous mastocytosis
	Erythema cronicum migrans
	Porokeratosis Mibelli
	Mycosis fungoides
	Vitiligo
	Annular lichenoid dermatitis of youth (ALDY)
Late stage *prevalent atrophy* *prevalent sclerosis* *prevalent hyperpigmentation*	
	Lichen sclerosus
	Morpheaform injection-site reactions
	Scarring
	Acrodermatitis chronica atrophicans

	Lipodermatosclerosis
	Carcinoma en cuirasse
	Sclerosing congenital melanocytic nevus/ connective tissue nevi
	Morpheaform basal cell carcinoma
	Necrobiosis lipoidica
	Pretibial myxedema

	Postinflammatory hyperpigmentation
	Erythema discromicum perstans
	Cafe-au-lait spots
Generalized morphea	
	Systemic sclerosis (scleroderma)
	Scleredema
	Scleromyxedema
	Chronic graft-versus-host disease
	Porphyria cutanea tarda
	Primary systemic amyloidosis
	Nephrogenic systemic fibrosis
	Morpheaform sarcoidosis
	Paraneoplastic scleroderma-like syndromes (e.g., POEMS syndrome)
	Phenylketonuria
	Genetic disorders
Linear morphea	
	Panniculitis
	Localized lipodystrophy
	Steroid-induced atrophy
	Sclerosing nevus
	Lupus erythematosus profundus
	Focal dermal hypoplasia
	Reflex sympathetic dystrophy
	Diabetic cheiroartropathy
	Eosinophilic fasciitis
Deep morphea	
	Panniculitis	Scleredema	Lipodystrophy	Eosinophilic fasciitis

## 6. Differential diagnosis

In the early disease stages, morphea can be challenging to differentiate from many inflammatory dermatoses, such as lichen sclerosus, granuloma annulare, erythema chronicum migrants or drug-induced dermatitis. All differential diagnoses that should be taken into consideration when diagnosing morphea are listed in Table 3.

**Table 3:** Clinical trials in morphea.

Study	Design	Intervention	Mechanism of drug	Outcomes	Phase	Status
**Topical treatments**						
NCT03351114	pilot, proof-of-concept, open label, single arm	Crisaborole 2% ointment, applied twice per day for 12 weeks	Topical PDE4 inhibitor	Primary outcome measures Change in dermal thickness on 4 mm skin punch biopsy Secondary outcome measures • reduction in DIET (dyspigmentation, induration, erythema, telangiectasias) score • reduction in LoSCAT (Localized Scleroderma Cutaneous Assessment Tool) score • reduction in Skindex-29 score (health-related quality of life) • change in dermal thickness of sentinel plaque (using ultrasonography)	2	Completed, awaiting results (2020)
NCT02411643	open label, single group assigned trial	Calcipotriene 0.005% ointment, applied twice per day for 3 months	Vitamin D analogue – anti-proliferative, anti-inflammatory	**Primary outcome measures** Change of gene expression from skin biopsy **Secondary outcome measure** • quality of life • modified Localized Scleroderma Skin Score • change of appearance of skin biopsy	1	Terminated (2018)
NCT00147771	non-randomized, single group open-label trial	Imiquimod 5% cream, applied 3-5x/week for 24 weeks	Toll-like receptor 7 agonist	**Primary outcome measures** Percent improvement in the skin thickness **Secondary outcome measures** Frequency of side-effects	3	Completed, awaiting results (2009)
**Phototherapy-based treatments**						
NCT04922736	non-randomized, non-blinded, open label, single group assigned trial	UVA1 phototherapy, total of 30 sessions	UV-mediated immunosuppression	**Primary outcome** **measures** Change in the Health Assessment Questionnaire Disability Index (HAQ-DI) after 30 sessions **Secondary outcome** **measures** Changes after 30 sessions in: • Hand Mobility in Scleroderma (HAMIS) score in patients with hand involvement • Localized Scleroderma Assessment Tool (LoSCAT) score durometer scores	N/A	Enrolling by invitation (2021)
NCT04954573	non-randomized, parallel assigned, open-label trial	Radiation: infrared-A Local-water filtered infrared-A irradiation	Acute and chronic wound healing and anti-inflammatory effects	**Primary outcome** **measures** Intensity of skin sclerosis determined by a high-frequency ultrasound device with a 22 MHz applicator **Secondary outcome** **measures** • assessment of modified Rodnan Skin Score (mRSS) skin score, of skin hardness determined by durometer, and of range of motions as measured by the range of motions in the presence of contractures • patient’s satisfaction determined by Patients’ Global Impression of Change (PGIC) scale	N/A	Recruiting (2021)
NCT04752397	observational, case-only, prospective trial	Extracorporeal photopheresis	UV-mediated immunosuppression	**Primary outcome** **measures** Change in Modified Rodnan Skin score in 17 areas of the body at weeks 4 ± 2, 8 ± 2, 12 ± 2, 16 ± 2, 20 ± 2, and 24 ± 2 **Secondary outcome** **measures** Change at lesional and control skin area at weeks 12 ± 2, and 24 ± 2 in: • skin thickness • transepidermal water loss (TEWL) • stratum corneum hydration (SCH) • skin firmness • skin surface sebum level Change at time point 3 (after completion of the cycle) in: • serum levels of proinflammatory factors Interleukin 4 (IL-4), Interleukin 9 (IL-9), Interleukin 33 (IL-33) and Transforming growth factor beta (TGF-beta) • serum levels of Platelet factor 4 (CXCL4) Acute change between time points 1 and 2 at weeks 8 ± 2, 16 ± 2, 24 ± 2 in: • serum levels of proinflammatory factors IL-4, IL-9, IL-33 and TGF-beta • serum levels of CXCL4 • percentage counts of Th1, Th2, Th17 and Treg cells Change between time points 1 and 3 after ECP cycle at weeks 8 ± 2, 16 ± 2, and 24 ± 2 in: • serum levels of proinflammatory factors IL-4, IL-9, IL-33 and TGF-beta • serum levels of CXCL4 • percentage counts of Th1, Th2, Th17 and Treg cells Change at time point 3 at weeks 8 ± 2, 16 ± 2, and 24 ± 2 in: • serum levels of proinflammatory factors IL-4, IL-9, IL-33 and TGF-beta • serum levels of CXCL4 • percentage counts of Th1, Th2, Th17 and Treg cells	N/S	Recruiting (2021)
NCT04875078	randomized, single-blind, cross over, parallel assigned trial	High dose (80-120 J/cm2) UVA1 phototherapy on one hand only vs. untreated hand covered with gloves, for a total of 30 sessions	UV-mediated immunosuppression	**Primary outcome** **measures** HAMIS score of treated hand compared to the untreated hand after 30 UVA1 treatments over approximately 100 days **Secondary outcome** **measures** Change from baseline to after 30 UVA-1 treatments of treated hand in: • HAMIS score • CHFDS score • skin hardness based on a durometer • skin thickness based on the modified Rodnan skin score (mRSS) • Skindex-16 score • Michigan Hand Questionnaire (MHQ) • Hand Disability in Systemic Sclerosis—Digital Ulcers (HDISS-DU) PROMIS Physical Function (PROMIS-PF)	N/A	Recruiting (2020)
NCT01799174	randomized, triple blinded, placebo controlled, parallel assignment trial	UVA1 (70 J/cm2) vs. placebo (0 J/cm2), applied 3×/week for 10 weeks	UV-mediated immunosuppression	**Primary outcome** **measures** Change in LoSSI from baseline vs. after 30 treatments **Secondary outcome** **measures** • Physician’s Global Assessment of disease Activity (PGA-A) over 3 years • Gene expression profiling over 3 years	N/A	Completed (2019)
NCT00812188	randomized, single blinded (investigator)	Fluocinonide 0.05% cream twice per day to one plaque for 12 weeks, and UVA-1 medium dose (60 J/cm2) or high dose (120 J/cm2), 3×/week for 12 weeks to another plaque	Corticosteroid-associated anti-inflammatory effects vs. UV-mediated immunosuppression	**Primary outcome** **measures** Efficacy of UVA-1 treatment versus topical steroid over a time frame of 5 years	N/A	Completed (2019)
NCT00476801	randomized, outcomes assessor, crossover assigned trial	UVA1 phototherapy, applied 5×/week for up to 14 weeks with dose increasing up to 130 J/cm2 on one side of the face vs. no treatment on opposite side, then cross-over treatment an equal length of time	UV-mediated immunosuppression	**Primary outcome** **measures** Plaque thickness and hardness, and increase in mobility at week 28 **Secondary outcome** **measures** Analysis of collagen levels and MMP induction at week 28	N/A	Completed (2004)
NCT00476697	open label, single group assigned trial	UVA1 phototherapy, applied 5×/week for up to 16 weeks with dose increasing up to 130 J/cm2	UV-mediated immunosuppression	**Primary outcome** **measures** Plaque thickness and hardness, and increase in mobility at week 16 **Secondary outcome** **measures** Analysis of collagen levels and MMP induction at week 16	N/A	Terminated (2003)
**Systemic treatments**						
NCT03740724	open label, single group assigned, trial	FCX-013 injected intradermally 1–2 times (12 weeks apart) + veledimex initiated on the day of injection and continued for 2 weeks	FCX-013 is a genetically modified autologous fibroblast that expresses metalloproteinase-1 under the control of a RheoSwitch induced by veledimex molecule	**Primary outcome** **measures** Safety **Secondary outcome** **measures** Evaluate the antifibrotic effects of FCX-013 plus veledimex	1/2	Terminated (2022)
NCT04200755	randomized, multi-center, double-blind, placebo controlled, parallel group trial	Dupixent (dupilumab) 300 mg (30 patients) vs. placebo (15 patients), first dose 2 s.c. injections, followed by 1 s.c. injections every 2 weeks for 24 weeks	IL-4/IL-13 inhibitor	**Primary outcome measures** Change in LoSCAT score of target lesion (from baseline to end of treatment visit, 24 weeks) **Secondary outcome measures** From baseline to follow-up visit, 48 weeks • change in mLoSSI (Localized Scleroderma Skin Activity Index), LoSDI (Localized Scleroderma Skin Damage Index), DermatoLogy Quality of life Index (DLQI) • number of lesions • adverse events • clinical parameters: physical examination, body weight, blood pressure, pulse rate, body temperature, number of lesions • laboratory parameters: hematocrit, hemoglobin, blood cell count, blood enzymes, clinical chemistry, antinuclear antibodies, serum cytokine levels From baseline to follow-up visit, 24 weeks • RNAseq data • RT-Qpcr data	2	Recruiting (2020)
NCT03388255	open label, single group assigned trial	Polydeoxyribonucleotide (PLACENTEX^®^) 5.625 mg/3 ml, daily i.m. injections for 3 months	unknown	**Primary outcome** **measures** Localized Scleroderma Cutaneous Assessment Tool—LOSCAT**Secondary outcome** **measures** Change in: • tele-thermographic profile (24 weeks) • ultrasound profile of target cutaneous lesion (24 weeks) measurement of histology improvement (12 weeks) • DLQI (24 weeks)	4	Terminated (2019)
NCT00936546	non-randomized, open label, single group assigned trial	Mabthera (rituximab) 1,000 mg injected i.v. at baseline and at 6 months	Anti-CD20	**Primary outcome** **measures** Safety at baseline, months 3, 6, 12, 15, 18, 24, 36, 48, and 60 **Secondary outcome** **measures** Efficacy at baseline, months 3, 6, 12, 15, 18, 24, 36, 48, and 60	2	Completed (2015)
NCT00479934	randomized, double blinded, placebo controlled, parallel assigned trial	Imatinib mesylate 400 mg/day *per os* vs. placebo for 6 months	Bcr-abl tyrosine kinase inhibitor	**Primary outcome** **measures** Percent variation of modified Rodnan score between inclusion and 6-month visits **Secondary outcome** **measures** • percent variation of modified Rodnan score between inclusion and follow-ups at 1, 3, and 12 months • skin thickness at inclusion and at 6 months using skin biopsies • quality of life using DLQI (Dermatology Quality of Life Index) and HAQ (Health Assessment Questionnaire) at 1, 3, 6, and 12 months • tolerance of treatment • effects of treatment on non-cutaneous symptoms	2	Completed (2010)
NCT00501995	open label, single site, single group assigned trial	Cyclophosphamide (50 mg/kg) i.v. daily for 4 consecutive days	DNA alkylating agent	6 patients started treatment, one patient died during the early phase. **Primary outcome measures** Improvement in mRSS from baseline (measured at 0, 1, 3, 6, 12, and 24 months; > 25% is considered significant): 46.75% improvement from baseline **Secondary outcome** **measures** Change in: • HAQ-DI (The Health Assessment Questionnaire-Disability Index): 79% improvement from baseline • physician global assessment (PGA) which is a visual analogue: 71% improvement from baseline	3	Completed, with results (2008)

We conducted a search on clinicaltrials.gov on October 12th 2022 with the key words ‘morphea’ and ‘localized scleroderma’. We identified a total of 36 studies. Of them, we kept only those that were updated since 2020, namely 17 studies. We excluded older studies, those that had status ‘unknown’ or ‘withdrawn’, and also the ones that focused on registries. Numbers in brackets in the Status column indicate the last year in which the study was updated on clinicaltrials.gov.

In adults, morphea can present similar clinical features with:

### 6.1. Systemic sclerosis

Ruling out SSc is essential for the clinician when first diagnosing morphea. The presence of specific characteristics, including facial (mask-like facial appearance, beak-shaped nose, telangiectasias and microstomia), vascular (Raynaud’s phenomenon, sclerodactyly, pitting scars and digital ulcers), serological (positive anti-centromere or anti-Scl-70 antibodies) and inner organ involvement, support the diagnosis of SSc (4, 6).

### 6.2. Lichen sclerosus

Lichen sclerosus is an inflammatory disease that presents with white atrophic patches mainly in the genital area, but extragenital involvement is also possible. A prospective study of 76 morphea patients showed that genital lichen sclerosus is more frequent in morphea patients than in healthy controls (125). Furthermore, approximately 6% of morphea patients present genital and/or extragenital lichen sclerosus (126). Notably, the coexistence of morphea and lichen sclerosus was observed only in the limited and generalized types. Therefore, it remains unclear whether they are two different diseases occurring simultaneously or the characteristic lichen sclerosus lesions represent features of morphea.

### 6.3. Carcinoma en cuirasse

Indurated plaques involving the skin overlying the breasts may be indicative of an underlying breast tumor or less commonly of other neoplasms (127). In addition, they may also occur as a complication of radiotherapy (128).

### 6.4. Lipodermatosclerosis

Lipodermatosclerosis is a frequent complication associated with chronic venous insufficiency, which is characterized by a circumferential induration of the skin on the lower leg, with a distinctive appearance of an inverted champagne bottle.

In children, following conditions may be more frequently mistaken with morphea: connective tissue nevi, localized lipodystrophy at the injection site, inflammatory vitiligo, annular lichenoid dermatitis of youth (ALDY), hypopigmented mycosis fungoides, erythema cronicum migrans, cutaneous mastocytosis, café au lait spots and eosinophilic fasciitis.

## 7. Management

### 7.1. Topical therapy

#### 7.1.1. Topical corticosteroids

Topical corticosteroids of moderate to high potency are used for active, limited types of morphea. They should be applied once a day for a period of up to 3 months (95). If longer applications are needed, they should be given as interval therapy. In addition, under occlusion applications or intralesional steroids injected in the active margin can be tried by means of increasing their efficacy, particularly in recalcitrant cases of superficial and linear morphea. However, there are no clinical studies to date regarding the use of topical corticosteroids in morphea.

#### 7.1.2. Topical tacrolimus

Tacrolimus 0.1% ointment may also be used in active, plaque-type morphea. A double-blind, placebo-controlled pilot study showed that when applied twice daily for 12 weeks it significantly improved morphea lesions in matter of clinical feature scores and skin hardness (129).

#### 7.1.3. Topical vitamin D derivatives

Topical calcipotriene 0.005% and calcipotriol 0.005% ointments applied twice daily for 3 months, either alone or in combination with phototherapy represent a good therapeutic option for active, plaque-type and linear variants, particularly in childhood morphea or in cases that are refractory to topical corticosteroids (130, 131).

#### 7.1.4. Topical imiquimod

Imiquimod 5% cream can be used in both pediatric and adult plaque-type morphea. When applied for a total period of 9 months, it was shown to significantly reduce skin thickening and induration with minimal and well-tolerated side effects, except for one pediatric patient that required temporary discontinuation due to skin ulceration (132, 133). Moreover, the 2019 SHARE working group recommends its use also in selected cases of non-progressive or extended forms of other juvenile morphea types (29).

### 7.2. Systemic therapy

#### 7.2.1. Methotrexate

Methotrexate, either alone or in combination with systemic corticosteroids, is considered the drug of choice for the treatment of deep, generalized, pansclerotic or progressive linear morphea, particularly in the presence of extracutaneous manifestations, including *en coup de sabre*-associated epilepsy (95, 134). It is also the first-line of treatment in moderate-to-severe pediatric morphea (29, 135). Based on the recommendations of Childhood Arthritis and Rheumatology Research Alliance (CARRA), there are three different treatment regimens available for pediatric morphea: (i) methotrexate monotherapy, (ii) pulsed methotrexate and methylprednisolone administered intravenously, and (iii) pulsed methotrexate and prednisone administered orally (136).

Methotrexate can be administered either orally or with subcutaneous injections in doses ranging from 0.3 to 0.6 mg/kg/week (15 mg/m^2^/week) in children and 15–25 mg/week in adults. It is commonly combined over the first 3 months with systemic corticosteroids (intravenous methylprednisolone 30 mg/kg/day for three consecutive days per month or prednisone 1–2 mg/kg/day with subsequent gradual tapering) as bridge therapy (29, 137–142).

In a randomized, double-blind controlled study of 70 children with active morphea, it was shown that methotrexate was superior to prednisone in matters of decrease in computerized skin score rates, development of new lesions and thermography findings at month 12 (143). In addition, the prednisone-only group showed a three times higher risk of recurrence than the methotrexate group. However, approximately 15% of patients with pediatric morphea relapse at 2-year follow up after treatment with methotrexate (144). Potential relapse predictors are older age at onset and linear morphea of the limbs (145). Nevertheless, methotrexate treatment duration lasting at least 1 year before tapering is associated with prolonged remission after methotrexate cessation (139, 144). Moreover, low-dose treatments are safe and well-tolerated in the pediatric population, even with longer treatment durations (137–139, 141–144). Accordingly, the SHARE working group recommends the discontinuation of methotrexate only when the patient is in remission and off steroids for at least 1 year (29).

#### 7.2.2. Systemic corticosteroids

As previously mentioned, systemic corticosteroids are commonly used in combination with methotrexate in the treatment of active deep, linear or generalized morphea (137–140). In monotherapy, the only published study showed that they were effective and well-tolerated in morphea, in a dose ranging from 0.5 to 1 mg/kg/day. Favorable clinical effects were seen in the first 3 months of treatment. However, one-third of patients relapsed after finishing the treatment (146).

#### 7.2.3. Mycophenolate mofetil

Mycophenolate mofetil is reserved for patients that are refractory, intolerant or with contraindications to methotrexate and/or relapsing, severe cases (29, 95). Therefore, it is considered a second-line treatment for both pediatric and adult morphea. Three retrospective cohort studies totalizing 94 morphea patients demonstrated the clinical efficacy and favorable safety profile of mycophenolate mofetil (147–149).

In a recent retrospective study comparing 22 patients with pediatric morphea treated with mycophenolate mofetil versus 47 methotrexate-responders, Martini et al. (150) showed that there were no significant differences regarding relapse-free survival and efficacy between the two groups. In addition, mycophenolate mofetil had a good safety profile and the combination with methotrexate did not increase its efficacy, suggesting its potential use as a first-line treatment in severe, pediatric morphea patients. However, prospective clinical studies with larger cohorts are needed for confirmation.

#### 7.2.4. Miscellaneous

Other agents including cyclosporine, hydroxychloroquine, azathioprine, retinoids, intravenous immunoglobulins, rituximab and infliximab, have all been shown effective in various case reports of severe morphea (151–157). However, their routine use is pending more definitive evidence of efficacy. On the other hand, current evidence does not support the use of oral calcitriol, penicillamine or IFN-γ for the treatment of morphea (158–160).

### 7.3. Phototherapy-based therapies

Ultraviolet (UV) light was shown to modulate different proinflammatory cytokines, deplete Langerhans cells and T cells, as well as induce MMP in cutaneous lesions, thus exerting potential anti-inflammatory and anti-fibrotic effects (161–163). Longer wavelengths (320-400 nm) penetrate deeper in the dermis than do shorter ones (280–320 nm), making UVA-based therapies effective for deep morphea lesions, and UVB-based for thin, superficial cutaneous sclerosis. Nevertheless, the UV penetration does not extend beyond the dermis, making it ineffective for morphea with deep structure involvement.

Phototherapy options include psoralen plus UVA (PUVA), broadband UVA, UVA1, narrow-band UVB and extracorporeal photopheresis (163–168). In a randomized controlled study comparing low- and medium-UVA1, and narrow-band UVB phototherapy in 64 morphea patients, medium-dose UVA1 was superior in reducing sclerosis and it was also well-tolerated (169). UVA1 is usually performed 3–5 times a week for a minimum of 30 sessions. Nevertheless, about half of the patients treated with UVA1 experience relapses within 3 years after phototherapy (170). In this case, a second cycle or systemics may be considered. If UVA1 phototherapy is not available, broadband PUVA is an effective and safe therapeutic alternative (171).

In children, the use of phototherapy for the treatment of morphea is challenging (29, 95). In addition, it is limited by the need for prolonged maintenance sessions, which are associated with high cumulative dosage irradiations, hence the risk of skin aging and carcinogenesis (172, 173). Therefore, current recommendations suggest that PUVA therapy should be avoided in children (105). Recently, a systematic review has demonstrated that methotrexate is superior to phototherapy in children with morphea, particularly in severe cases (174).

Newer phototherapy-based therapies for morphea include laser therapy, with excimer laser being suitable for inflammatory lesions, whereas pulsed dye, alexandrite, Nd:YAG or fractional lasers are more effective for sclerotic and atrophic lesions (175). However, the majority of available data rely on case series and uncontrolled studies, and laser therapy is commonly used in combination with other treatments, thus making it hard to assess the real effectiveness of laser therapy. The only randomized controlled study comparing fractional laser with low-dose UVA1 phototherapy in 17 patients with linear or plaque-type morphea, has confirmed the higher efficacy of laser therapy in matters of clinical scores, histopathological (i.e., collagen homogenization) and ultrasound parameters (i.e., dermal thickness) (176). However, these findings need to be validated in larger cohorts as well as in comparison to different UV doses and other conventional methods.

### 7.4. Other measures

Beside pharmacologic therapy, the treatment of morphea may also include general measures, such as psychosocial support, physiotherapy, massage, lymphatic drainage, interdisciplinary consultations (rheumatology, physical medicine and rehabilitation, orthopedics, plastic and oral maxillofacial surgery) and surgery.

#### 7.4.1. Physiotherapy and massage

Physical therapy is indicated in all types of morphea that may result in limitations in range of motion, including linear, deep, generalized and mixed types. It is usually performed 1–2 times weekly for at least 3 months and should be avoided in the active disease stage. Massage and lymphatic drainage can also be done, particularly in the sclerotic stage (95).

#### 7.4.2. Surgical therapy

Orthopedic surgery may be needed in case of limb-length discrepancy, the latter being common in linear morphea of the limbs, but also in deep and generalized variants (177). In case of linear *en coup de sabre* morphea or Parry-Romberg syndrome, facial deformities can be corrected with plastic-surgical interventions (178). To minimize the risk of disease reactivations, surgery should only be performed when the disease is in remission (95, 105).

Newer cosmetic surgeries include bone paste cranioplasty, Medpor implants for facial deformities and autologous fat injection (178, 179). The latter has been shown to exert also anti-inflammatory and anti-fibrotic effects due to the presence of adipose stem cells in the processed tissue (180, 181). In addition, autologous fat injections may be performed to a certain extent even in the active disease stage as well as in pediatric morphea. All these therapies may be used as an adjunct to systemic therapies in order to improve cosmetic, physical and functional outcomes.

### 7.5. Emerging therapies

Despite numerous available therapeutics, the treatment of morphea still remains a challenge. Current treatment is not-disease specific and its long-term use is associated with significant morbidity. In addition, it was shown that certain patients experience relapses after therapy cessation, while others are refractory to the most common treatment options (13, 16, 144–146, 148, 170). In light of this, recent advances in our understanding of the pathogenesis of morphea have identified various potential therapeutic targets. Current clinical trials in morphea are shown in Table 3.

#### 7.5.1. Anti-fibrotic drugs

Despite the not completely understood pathophysiology of morphea, it seems that inflammatory and profibrotic processes are mediated mainly through the TGF-ß and PDGF pathways (11). Imatinib, a tyrosine kinase inhibitor that interferes with both signaling pathways by blocking the activity of c-Abl, c-Kit and PDGF receptors, respectively, showed beneficial results in numerous case reports of morphea patients (182–185). In addition, there is an ongoing phase 2 randomized clinical trial (NCT00479934) in morphea, with results pending.

Connective tissue growth factor (CTGF) is a profibrotic peptide that acts downstream of TGF-ß and is highly expressed in morphea lesional skin (186). Interestingly, iloprost, a prostaglandin analogue, which is already used in SSc patients for the treatment of severe Raynaud’s syndrome, can suppress the secretion of CTGF by fibroblasts (187). Furthermore, a randomized clinical trial with the anti-CTGF monoclonal antibody, pamrevlumab, demonstrated its favorable effect in reducing disease progression of idiopathic pulmonary fibrosis (188). Based on these findings, morphea patients could also benefit from prostaglandin analogues as well as anti-CTGF biologics.

#### 7.5.2. Anti-inflammatory drugs

IL-6 plays a crucial role in the pathogenesis of morphea. It exerts both inflammatory and profibrotic effects by binding to its membrane receptor (IL-6R) and activating the downstream Janus kinase (JAK)-STAT. The latter leads to the stimulation of collagen and MMP production by fibroblasts, and the differentiation of naïve CD4+ to pathogenic T_H_17 cells *via* the putative TGF-ß axis (189, 190). Accordingly, IL-6 was shown to be increased in both sera and lesional skin of morphea patients (9, 191). Tocilizumab, a fully humanized antibody against IL-6R, demonstrated promising results in three case series totalizing 8 children with pansclerotic as well as with deep morphea, including joint involvement (192–194). Another IL-6R antibody, sarilumab, was evaluated in a phase 2, open-label clinical trial, which, however, has been recently withdrawn due to difficulty in recruiting patients (NCT03679845). Further controlled clinical studies are needed to evaluate the therapeutic potential of these biologics in morphea.

Another possible target is the JAK/STAT signaling pathway, which acts downstream of the central TGF-ß axis. Recent *in vitro* and murine studies showed that JAK inhibitors were able to successfully block the TGF-ß-driven skin fibrosis (195–197). In fact, tofacitinib, a JAK1 and JAK3 inhibitor, led to improvement in both clinical and histological skin thickness and also joint mobility in numerous cases of refractory, generalized morphea (197–199). Therapeutic response was observed after first month, with a maximum between 11 and 16 months, while improvement was still noted even at month 30 after treatment initiation, this without any major side effects. Similarly, baricitinib, a JAK1 and JAK2 inhibitor, showed positive effects in one patient with generalized morphea (197). In contrast, ruxolitinib, another JAK1 and JAK2 inhibitor, failed to control the disease progression in a child with refractory, pansclerotic morphea (200). Larger controlled studies need to validate these findings.

Abatacept, a soluble recombinant cytotoxic T-lymphocyte-associated protein 4 fusion protein has gained recent attention as a potential novel therapeutic option in severe morphea. Studies showed that it cannot only prevent but also limit dermal fibrosis in various mouse models of SSc (201). Moreover, many clinical case series and cohort studies of both pediatric and adult patients proved its effectivity in severe, refractory and/or deep morphea (202–204). In addition, approximately 80% of patients receiving abatacept were responders at month 12 in matters of both cutaneous and musculoskeletal activity (205). However, 16.7% had to discontinue the treatment due to adverse reactions. Still, abatacept may be a good therapeutic option in patients with severe morphea that are refractory to conventional treatment.

Autologous stem cell transplantation is another possible therapeutic option in morphea, in particular for severe cases that are refractory to current available therapies. Two recent case reports of disabling pansclerotic morphea of childhood demonstrated its beneficial effect (206, 207). However, without any concomitant systemic therapy, the disease relapsed after treatment termination.

## 8. Clinical course and prognosis

Although morphea is rarely life-threatening, the disease is characterized by a chronic, relapsing–remitting course, which can cause a lot of disease burden over time. Furthermore, in certain types it may be associated with extracutaneous manifestations that may lead to functional impairments, cosmetic disfigurements, psychological stress as well as significant decrease in patients’ quality of life (13–17).

Recurrences occur in approximately one-quarter of morphea patients (13). Relapse rates were more frequent in children than in adult morphea, namely 27% and 17%, respectively. Risk factors for relapse include age of onset, disease type (particularly linear morphea of the extremities, but also the generalized type), delay in starting treatment and the presence of positive ANA titers (13, 17, 145). A median duration of 26 months between disease remission and first relapse was described in juvenile morphea, similar to the adult form (13). Nevertheless, the active disease duration was much longer in childhood morphea, whereas certain patients present longer times of disease remission before experiencing a relapse, thus underscoring the need for longer follow-up periods (14, 208). Since linear morphea is more frequent in children and the disease course is more severe than in adults with a higher risk of complications and functional damage (7, 14, 16, 26, 208), close and multidisciplinary follow-up is crucial. In addition, the delay of treatment was associated with higher rates of relapse as well as higher disease activity (17). Therefore, children diagnosed with morphea require prompt initiation of systemic treatment and close longer follow-ups, particularly in the first 2 years after treatment discontinuation.

## 9. Conclusion

Disease activity assessment based on current validated clinical scores is a crucial step in the initial evaluation of patients with morphea. A late diagnosis or an incorrect severity assessment may lead to a delay of appropriate treatment, and thus to physical and functional disabilities as well as decreased quality of life. This applies in particular to pediatric morphea, especially the linear and deep types, where initiating adequate systemics is pivotal for achieving disease control and reducing subsequent damage. In addition, childhood morphea is associated with a more severe disease course and higher risk of relapse, the latter occurring even after years of remission. Moreover, certain cases are refractory even to current therapeutics, i.e., methotrexate, systemic corticosteroids and mycophenolate mofetil. On the other hand, recent advances in our understanding of the pathophysiology of morphea identified novel targets that may be used to inhibit the early inflammatory processes so as to impede fibrosis and atrophic changes. Still, the disease may require combination therapies as well as long follow-ups.

## Author contributions

All authors listed have made a substantial, direct, and intellectual contribution to the work and approved it for publication.

## Funding

This research was supported by the Schleswig-Holstein Excellence-Chair Program from the State of Schleswig Holstein as well as by DFG: Excellence Cluster EXC 2167 *Precision Medicine in Chronic Inflammation* and the Research Training Group *Autoimmune Pre-Disease* (GRK 2633).

## Conflict of interest

The authors declare that the research was conducted in the absence of any commercial or financial relationships that could be construed as a potential conflict of interest.

## Publisher’s note

All claims expressed in this article are solely those of the authors and do not necessarily represent those of their affiliated organizations, or those of the publisher, the editors and the reviewers. Any product that may be evaluated in this article, or claim that may be made by its manufacturer, is not guaranteed or endorsed by the publisher.
